# Validity and Reliability of Cultural Mix Coping Inventory for Stressful Situations among Healthcare Professionals in Ghana Amidst COVID-19

**DOI:** 10.3390/ijerph191710651

**Published:** 2022-08-26

**Authors:** Francis Ankomah, Frank Quansah, Edmond Kwesi Agormedah, John Elvis Hagan, Medina Srem-Sai, Francis Sambah, Abdul-Aziz Seidu, Edward Kwabena Ameyaw, Bright Opoku Ahinkorah, Eugene Kofuor Maafo Darteh, Thomas Schack

**Affiliations:** 1 Department of Education and Psychology, University of Cape Coast, Cape Coast PMB TF0494, Ghana; 2 Department of Education, SDA College of Education, Asokore-Koforidua P.O. Box AS 18, Ghana; 3 Department of Educational Foundations, University of Education, Winneba P.O. Box 25, Ghana; 4 Department of Business & Social Sciences Education, University of Cape Coast, Cape Coast PMB TF0494, Ghana; 5 Department of Health, Physical Education and Recreation, University of Cape Coast, Cape Coast PMB TF0494, Ghana; 6 Neurocognition and Action-Biomechanics-Research Group, Faculty of Psychology and Sports Science, Bielefeld University, Postfach 10 01 31, 33501 Bielefeld, Germany; 7 Department of Health, Physical Education, Recreation and Sports, University of Education, Winneba P.O. Box 25, Ghana; 8 College of Public Health, Medical and Veterinary Sciences, James Cook University, Townsville, QLD 4811, Australia; 9 Centre for Gender and Advocacy, Takoradi Technical University, Takoradi P.O. Box 256, Ghana; 10 School of Public Health, Faculty of Health, University of Technology Sydney, Sydney, NSW 2007, Australia; 11 Department of Population and Health, University of Cape Coast, Cape Coast PMB TF0494, Ghana

**Keywords:** coping inventory, culture, Ghana, health workers, reliability, stressful situation, validity

## Abstract

The Cultural Mix Coping Inventory for Stressful Situations is one of the recent coping measures developed to overcome the weaknesses of existing coping scales. Since its development and validation, the inventory has been used by previous studies to measure coping among teachers and students in stressful situations. Health professionals are workers who typically encounter stressful situations due to their work demands. In this study, we assessed the validity and reliability of cultural mix inventory for stressful situations among healthcare professionals in Ghana. The research was guided by three major objectives: (1) to assess the factor structure of the cultural mix coping inventory, (2) to evaluate the construct validity and reliability of the cultural mix coping inventory based on internal structure and (3) to test for evidence of criterion validity based on the external structure of the measure. Approximately 312 health workers were purposefully sampled to participate in the study. The study confirmed the original four-factor solution of the coping inventory with evidence of the construct validity based on the internal structure. Validity evidence based on the external structure of the measure was found to be sufficient. Given the COVID-19 pandemic and coupled with the stressful nature in the line of duty of healthcare professionals, this inventory provides a useful and sound measure of coping options among this cohort.

## 1. Introduction

Healthcare professionals, especially those who acted as frontline workers during the COVID-19 pandemic, were highly vulnerable to stress among all occupational groups due to the nature of their work environment [1,2,3]. The level of stress among healthcare professionals was intensified with increasing rates of coronavirus infection and fatality leading to mental health vulnerabilities [4,5,6,7]. Previous studies prior to the COVID-19 pandemic discovered that healthcare workers experience a high level of occupational stress as a result of scarce resources, heavy workloads, extended working hours, inadequate staffing, time-related pressure and vulnerability to contracting ailments from patients with communicable diseases [8,9]. Substantial evidence have established that healthcare workers experienced moderate-to-extremely-severe symptoms of stress, anxiety and depression during the coronavirus pandemic period [10,11,12]. These mental health and psychological distress challenges of healthcare professionals could cause significant impairment in their quality and efficiency (i.e., productivity of work), physical and emotional well-being, reduce job satisfaction and quality of care given to patients and increase the incidence of medical errors and accidents [13,14,15,16].

Healthcare professionals’ perception of stress depends on a cognitive evaluation process to evaluate the significance of events and also the potential coping resources to deal with them. To deal with stressful life events, individuals (including healthcare professionals) use a wide range of coping strategies [17,18]. Lazarus [17] identified two distinct functions of coping: problem-focused coping, which aims to actively change the stressful environment (i.e., efforts towards directly doing something to lessen the stressful event); and emotion-focused coping, which deals with strategies that aim at reducing emotional distress or expressing emotions, such as engaging in distracting activities and venting emotions (i.e., efforts towards dealing with their emotions as a result of the stressful situation) [19]. According to Lazarus [17], both problem-focused and emotion-focused coping can be adaptive at various times, based on the demands of a situation.

Given the significant role played by coping strategies in moderating the effect of stress in diverse groups, several instruments have been developed and psychometrically validated to measure the coping strategies adopted by individuals in stressful situations in different jurisdictions. Examples of these measures include a coping scale for caregivers in Malaysia [20], coping strategies in relatives of patients with schizophrenia in Italy [21], a situated coping questionnaire for adults in Spain [22], a brief approach/avoidance coping questionnaire for primary care patients in Norway [23], coping for competitive athletes in Canada [24], a coping with stress scale for children and adolescents in Turkey [25] and Canada [26], coping among students in France [27] and Canada [28] and the interpersonal stress coping scale in Japan [29]. 

Other generic coping inventories widely adopted are the coping strategies questionnaire [CSQ] [30], the coping resource inventory [31], the cope questionnaire [32] and the coping inventory for stressful situations [CISS] [33]. However, most of these existing scales have ignored culturally dominant codes, such as religion, except for a few isolated religious coping scales developed by different authors [34,35,36,37]. Culture plays a pivotal role in an individual’s coping strategy, especially in terms of collectivistic versus individualistic cultures [38,39]. Taking the individualism (self) and collectivism (in-group) theory, for example, coping strategies tend to be different depending on one’s cultural orientation and belief system [40]. While people from individualistic cultures are likely to focus on adapting to external stressors and largely utilize problem-focused strategies, the collectivistic culture, on the other hand, is oriented towards modifying oneself through the use of emotion and avoidance coping strategies [41]. As culture affects norms [42], this may affect coping behaviour through social norms [39,43]. Under the COVID-19 pandemic, the culture may largely define what uncertainty is (i.e., the perceived difficulty of a situation) and how it is dealt with, and this shapes the ways people cope with anxiety [39,44]. Religion is a key component of culture, and these two are inseparable [45]. Accordingly, coping inventories/scales that do not pay attention to cultural issues, such as religion, miss an important cultural element (i.e., diversity), especially in societies where cultural issues are dominant [46].

Based on the limitation of the existing coping scales and the relevance of culture (i.e., religion), a new instrument called the “Cultural Mix Coping Inventory for Stressful Situations” was developed in Ghana by Quansah et al. [46]. The newly developed cultural mix coping inventory is a 16-item multi-dimensional instrument with four subscales—namely, active coping, behaviour disengagement, religious coping and emotional support. The inventory has each dimension with four items aimed at measuring different forms of coping mechanisms. The scale provides satisfactory model fit, construct validity and convergent validity. The McDonald’s Omega reliability coefficients for the active coping, religious coping, behaviour disengagement and emotional support dimensions are 0.82, 0.81, 0.87 and 0.83, respectively. Since the development and calibration of the instrument, it has been adopted as a coping measure in recent studies to assess how teachers and students cope with varied stressful experiences [47,48,49,50,51].

From previous empirical evidence, the cultural mix coping inventory is worth re-validating using different samples (e.g., healthcare professionals who experience stressful situations in this pandemic period). Additionally, it is the only coping inventory developed in Ghana and happens to be among the few coping scales that have a mix of religion and emotional support together with other conventional coping strategies. Equally, the authors of the original scale [46] intimated that the coping inventory is flexible because the items are not specific to a particular context. Hence, it could be useful for assessing stressful experiences of other study populations (e.g., pre-tertiary students, teachers and healthcare professionals). According to Quansah and partners [46], the original validation study is not exhaustive, and thus further validation is needed to strongly establish its usefulness and applicability in other contexts using different samples.

For numerous reasons, healthcare professionals in Ghana were chosen as the normative group and special population of interest in this re-validation study because they typically encounter high levels of stressful experiences in the line of their duties [52,53]. For instance, healthcare professionals experienced heightened stress levels from the onset of the COVID-19 epidemic as frontline workers due to a dramatic shift in the regular ways of living and job routines in Ghana [54,55,56]. The lack of preparedness, inadequate working conditions, low healthcare worker: patient ratios (i.e., staff shortages), lack of personal protective equipment, limited testing capacity, lack of training and excessive workloads further exacerbated healthcare professionals’ stressful experiences [2,52,53,54,55,56]. The overall aim of this research, therefore, was to assess the validity and reliability of the cultural mix inventory for stressful situations among healthcare professionals in Ghana. The research was guided by three major objectives: (1) to confirm the factor structure of the cultural mix coping inventory, (2) to evaluate the construct validity and reliability of the cultural mix coping inventory based on the internal structure and (3) to test for evidence of the criterion validity by correlating the coping measure scores with anxiety scores (based on the external structure). 

The internal structure of the scale reflects the extent to which the relationship among the items on a scale is consistent with the proposed score interpretation of the inventory [57]. Further, the study conceptualizes the external structure as the degree to which scores from the inventory are related to other external variables under consideration [57]. In the context of this research, evidence from statistical parameters, such as variances, item loadings, reliability coefficients and measurement invariance analysis are used to judge the validity evidence based on the internal structure. In relation to the external structure, scores from a standardized anxiety scale were correlated with scores from the coping measure. This research makes substantial strides regarding the discussions on coping measurement and measures in the psychological testing literature. The study continues the discourse on widening the applicability, use and utility of the Cultural Mix Coping Inventory for Stressful Inventory among diverse samples. Thus, assessing the applicability of the coping inventory using healthcare workers compared to university students in the original development and calibration study would provide empirical evidence to support the use and utility of the inventory in healthcare workers. 

Additionally, the approach to the validation of this inventory using healthcare professionals adopted in this study differed from the calibration procedure used in the original validation. For instance, different competing models were identified and compared with the original four-factor structure model. The external structure of the coping measure was assessed by examining how the dimensions confirmed are associated with anxiety measures; this was not explored in the original validation study. These validation strategies adopted in this research support current developments in validity theory, which strongly recommends studying and combining multiple sources of validity evidence into a validity argument to support the use and interpretations of scores from the measure [58].

## 2. Methods and Materials

### 2.1. Study Participants

The study covers healthcare professionals, including nurses, midwives, surgical doctors, physician assistants and psychologists within the healthcare system who provided treatment services during the COVID-19 pandemic in Ghana. An initial sample projection of 300 was considered based on the recommendations of Tabachnick and Fidell [59] who suggested that a sample of 300 is appropriate for a Confirmatory Factor Analysis (CFA) with three to four dimensions. Further, a 10% non-response was computed based on Tabachnick and Fidell’s [59] suggestion which resulted in a sample of 330; however, only 312 participants responded to the survey instrument through the purposive sampling technique, reflecting a return rate of 94.5%. The participants had the following demographic information: sex (male or female), religion (Christian, Muslim; Traditionalist or Atheist), age, educational level (certificate, diploma, bachelor’s, master’s or PhD), job designation (general nurse, psychologist, physician assistant, surgical doctor or midwife) and years of working (see Table 1 for details).

### 2.2. Data Collection Instrument

A questionnaire was designed to collect the data for this research. The instrument had three main sections. The first section contained demographic information about the respondents, which included sex, religion, age, education qualification, job designation and years of working in the health sector. The second section had measures on Cultural Mix Coping Inventory for Stressful Situations, whereas the last segment of the instrument had items measuring anxiety. The scales used as measures for coping and anxiety are described in a subsequent section of the paper.

#### 2.2.1. Cultural Mix Coping Inventory for Stressful Situations

The cultural mix coping inventory for stressful situations is a multi-dimensional 16-item instrument used for assessing the coping strategies of any target group who is in stressful situations [46]. The inventory has four sub-scales—namely, active coping, religious coping, behaviour disengagement and emotional support. Each sub-category of the scale has four items measured on a four-point Likert type scale (not adopted = 1, somewhat adopted = 2, much adopted = 3 and very much adopted = 4). The participants were required to indicate the strategies adopted when in stress-induced situations, such as taking care of patients with COVID-19. The inventory has a high level of construct validity with the reliability coefficient of the sub-scales ranging from 0.812 to 0.869 [46]. The scale has also been found to function equally for both male and female respondents [46].

#### 2.2.2. Anxiety

The anxiety scale developed by Beck et al. [50] was adapted to measure the anxiety experiences of healthcare professionals when attending to patients in their workplace. The adaptation reflects using only the non-clinical proxies of Beck et al.’s scale since the respondents in this study were not participants diagnosed with any clinical conditions. Six items were adopted with response options 0 to 3 (0—“not at all”, 1—somewhat, 2—“moderately” and 3—“very much so”). The healthcare workers were expected to report their anxiety responses when caring for patients with COVID-19 virus. The listed items include: “I fear the worst happening”, “I feel unsteady”, “I feel very much concerned”, “I feel nervous”, “I have self-doubts” and “I feel unrelaxed”. The original anxiety scale has high reliability (Cronbach alpha) and validity indices (convergent and discriminant validity using correlational measures) [60]. This study reported a reliability coefficient of 0.831 for the anxiety scale using the McDonald’s Omega reliability estimation approach. This approach to the measurement of anxiety has been adopted by other previous studies, and sufficient reliability and validity have been provided [61].

### 2.3. Procedure and Ethics

Ethical approval for the study was sought from the Institutional Review Board (IRB) of the University of Cape Coast with the approval number UCCIRB/EXT/2020/25. Afterwards, contacts were made with hospital administrators and those in charge of the disease control departments. The eligibility criteria for participation were solely healthcare professionals who took care of COVID-19 patients (or suspected cases of COVID-19), including other certified professionals who cared for patients with non-communicable and communicable diseases. The emails and phone contacts of such qualified health care professionals were obtained and later contacted to seek their consent or willingness to participate in the study. Additionally, the participants provided electronic informed consent before responding to the questionnaire. An online survey was conducted by sending the electronic questionnaire to the participants through emails and other platforms as preferred by the respondents. The data collection period spanned from December 2020 to September 2021.

### 2.4. Statistical Analysis

The data were screened for missing data, outliers and data entry errors. The descriptive statistics (i.e., the mean, standard deviation, skewness and kurtosis) of the items are presented. A series of varied CFA was performed to confirm the factorial structure of the coping inventory by comparing the original four-factor structure with three other ones (i.e., one-factor, three-factor and second-order CFA models). The competing models were carefully selected. For example, the three-factor model was chosen based on the associations among the sub-dimensions of the cultural mix coping inventory. In the original validation, the authors [46] revealed an inter-dimension correlation coefficient of 0.629 for behaviour disengagement and religious coping; hence, those domains were merged to obtain a three-factor solution. Deciding on the best fit model, several indices of the specified models were compared. 

Particularly, the model with the lowest Akaike’s Information Criteria (AIC) and Bayesian Information Criteria (BIC) was judged as the best. The following cut-offs were also used for other indicators used to evaluate the item characteristics of the four-factor structure CFA: chi-square, a non-significant *p* value, with χ^2^/df below 3.0; the Comparative Fit Index (CFI) > 0.90; the Root Mean Square Error of Approximation (RMSEA) < 0.10; and the Standardized Root Mean Square Residual (SRMR) < 0.08. Both the factor loadings and Average Variance Extracted (AVE) estimates were interpreted using a benchmark of 0.50 [62,63]. The Analysis of Moment Structure (AMOS, Version 21) was used for the data analyses. The reliability of the scale was assessed using the McDonald’s Omega ω approach. 

The heterotrait–monotrait (HTMT) ratio of correlation approach was used to assess the discriminant validity of the scale using 0.90 as the benchmark [64]. The McDonald’s Omega estimation method employs the congeneric assumption and estimates are computed based on item loadings [65]. Recent pieces of evidence have shown that the McDonald’s Omega procedure is superior to other reliability estimation methods and, thus, provides accurate reliability coefficients [66]. A multi-group CFA and model-level invariance analyses were performed to examine measurement invariance based on the sex of respondents. Validity evidence based on the external structure was examined using anxiety as the criterion measure through correlational analysis. The factor analyses were performed using the maximum likelihood estimation procedure. 

## 3. Results

### 3.1. Socio-Demographic Characteristics

The demographic information of the 312 health workers who participated in the study is presented in Table 1.

About two-thirds of the participants were female health workers (*n* = 198, 63.5%), with the rest being male participants (*n* = 114, 36.5%). Regarding religion, participants with Christian affiliation constituted over 90% of the sample (*n* = 286, 91.7%), 7.7% were Muslim (*n* = 24), and two identified themselves as atheists. The largest proportion of the participants was aged between 30 to 34 years (*n* = 108, 34.6%). The following educational qualifications were mentioned by the participants: Certificate (8.3%), Diploma (28.8%), Bachelor’s (31.4%), Master’s (26.3%) and PhD (5.1%). More than 60% of the health workers had 5 or more years of working in the health sector (61.5%).

### 3.2. Descriptive Analyses

The mean, variance, skewness and kurtosis of the items were explored as presented in Table 2.

The mean values/responses for the individual items ranged from 0.479 (BEH3, “*I give up the attempt in dealing with the stressor*”) to 2.212 (ACP4, “*I do what has to be done, one step at a time*”) with their respective variance of 0.569 and 0.633. The skewness of the data ranged from −0.492 to 1.519. Similarly, the kurtosis estimate also ranged from −1.115 to 1.247. Both the skewness and kurtosis values were within the acceptable ranges.

### 3.3. Confirming the Factorial Structure of the Coping Inventory

To confirm the factorial structure of the scale, we conducted four-factor CFA and compared the model fit indices with three other competing models: a Unidimensional model, a three-factor model and a second-order model. The outcome of the analysis is presented in Table 3.

The results revealed that the four-factor structure model best fits the data compared with the other proposed competing models (see Table 3). Taking the CFI index, for instance, the four-factor structure model had a value of 0.954 relative to 0.321 (one-factor model), 0.615 (three-factor model) and 0.426 for the second-order CFA model. Focusing on the model selection indices, the AIC and BIC indicators of the four-factor model showed the least values (AIC: 373.734 vs. 12,566, 11,888 and 2459.883; BIC: 515.968 vs. 12,746, 12,079 and 2519.771).

### 3.4. Construct Validity (Convergent and Divergent) and Reliability Based on the Internal Structure of the Scale

**Figure 1:**
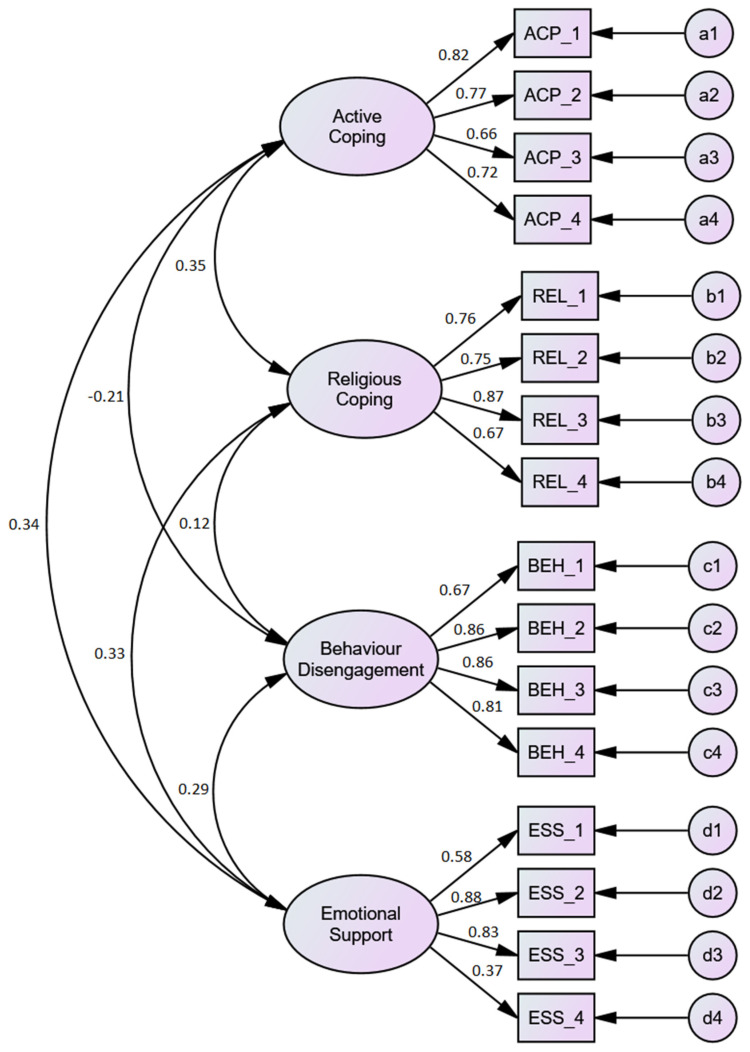
Multi-Factor First-Order CFA Model (Model 1).

The analysis output of the four-factor structure, 16-item CFA model is presented in Table 4 and Figure 1.

As presented in Table 4 and Figure 1, except for ESS4 “I learn to live with the stressor”, all the items had factor loadings greater than the 0.50 recommended benchmark as suggested by several scholars [62]. Taking the active coping dimension, for example, the factor loading ranged from 0.660 to 0.819, religious coping had loadings between 0.668 and 0.868, behaviour disengagement from 0.667 to 0.863, and emotional support had loadings from 0.373 to 0.873. 

Further, the AVEs of the sub-domains were also assessed. The values were within the acceptable range recommended by Fornell and Larcker [64] that the AVE should be greater than 0.50. All the sub-domains of the coping measure had an AVE larger than 0.50. Notably, the emotional support had an AVE of 0.666 although one of the items under the construct had low factor loading. That notwithstanding, the AVE values together with the loadings showed an adequate level of convergent validity. 

The results also revealed sufficient evidence of discriminant validity (based on internal structure). All the HTMT values were greater than 0.90, suggesting that divergent validity was established. Additionally, the reliability coefficients of the sub-dimensions were greater than 0.70 [63,65] with emotional support having the least (ω = 0.770) and behaviour disengagement showing the highest reliability index (ω = 0.873). The covariances among the sub-domains were also deemed appropriate (see Figure 1) [67]. 

#### Model 1 (Four-Factor, 16-Items) vs. Model 2 (Four-Factor, 15-Items)

Due to the low factor loading of ESS4, a new CFA model was fitted by deleting the item identified with the low loading. The new model (four-factor, 15 items) was compared with the original model (four-factor, 16 items) to assess whether deleting the ESS4 would provide a more valid model (see Table 5).

The outcome of the results, as shown in Table 5, revealed that there is a significant difference between the models, χ^2^(14) = 89.50, *p* < 0.001. A post hoc analysis further revealed that the original model (four-factor, 16 items) performed better than the modified model (four-factor, 15 items). For example, the AIC (224.388 vs. 309.888) and BIC (340.283 vs. 444.636) showed lower values for the original model when compared to the modified model.

### 3.5. Multi-Group Analysis Based on Sex

We conducted a multi-group analysis based on sex to examine whether male and female health workers had different results on the multi-factor first-order CFA model (i.e., the original specified model with 16 items). The details of the results are shown in Table 6.

The multi-group CFA revealed that male and female health workers were similar in terms of the measurement model, χ^2^(24) = 30.331, *p* = 0.174. Similarly, no statistical difference was found between male and female health workers regarding the structural covariances, χ^2^(44) = 74.279, *p* = 0.063. However, there was a significant difference between the two groups based on the measurement residuals, χ^2^(76) = 2.539, *p* < 0.001. 

Further, the model-level invariance performed revealed a non-significant difference in the chi-square test for the unconstrained versus the fully constrained model, χ^2^ (31) = 11.012, *p* = 0.116. Inspection of other model fit indices from the male and female models showed little difference between the models. The ∆RMSEA of 0.002, for one, adds to the evidence of invariance across sex. Similarly, ∆CFI and ∆SRMR values of 0.001 and 0, respectively, also confirmed the presence of measurement invariance across male and female healthcare workers.

### 3.6. Validity Evidence Based on the External Structure 

Validity evidence based on the external structure of the coping inventory was assessed using the correlation matrix. This analysis was done by examining the relationship the specific domains have with an anxiety measure. The outcome of the results is shown in Table 7.

The analysis presented in Table 7 reveals a significantly strong negative relationship between active coping and anxiety (*r* = −0.807, *p* < 0.001). A similar relationship was also found between religious coping and anxiety (*r* = −0.554, *p* < 0.001) as well as emotional support and anxiety (*r* = −0.807, *p* < 0.001). The result provides sufficient evidence to support the validity of the coping measure based on the external structure.

## 4. Discussion

The present study revalidated the cultural mix coping inventory for stressful situations by assessing its validity and reliability among healthcare professionals in Ghana. The objective of the research was in three-fold. First, the factor structure was evaluated by comparing the four-factor model with other competing models, such as one-factor, three-factor and second-order CFA models. Secondly, based on the internal structure as a source of validity evidence, the construct validity (i.e., convergent and divergent) and reliability were evaluated, and lastly, criterion validity evidence was established based on the external structure of the measure. 

The four-factor structure was confirmed as the best fit model after the model fit indices were compared with the one-factor, three-factor and second-order CFA models. These factors include active coping, religious coping, behaviour disengagement and emotional support. This finding means that, among healthcare professionals in Ghana, coping manifests in the form of active coping where several efforts are made to alter the stress-emanating situations: religious coping—being connected to a supernatural being or object of worship during stressful situations; behaviour disengagement—giving up or quitting to avoid stress; and emotional support—seeking support and empathy from friends or relatives. 

The four-factor structure revealed confirmed the original version of the inventory [46]. Generally, the four-factor confirmed in this study reflect the coping measures by other researchers [20,22,24,25,26,27]. For instance, Alonso-Tapia et al. [22] identified emotional expression and help-seeking as some of their dimensions. These dimensions relate to the emotional support aspect of the current validated coping inventory. Similarly, Kowalski and Crocker [26] considered emotion focus and avoidance coping as two of its three dimensions, and these relate to the emotional support and behaviour disengagement dimensions of the coping inventory under study. Further, Ibrahim et al. [20] equally identified religion and social support as coping dimensions, which are in line with the current coping inventory. In all, the implication is that the cultural mix coping inventory is culturally relevant and also includes several components of other coping inventories developed in other contexts. The specific characteristics of the items/sub-scales of the confirmed four-factor structure of the cultural mix coping inventory were further inspected. 

However, for one item (ESS4, “I learn to live with the stressor”), all the items had factor loadings more than 0.50, implying that each of the items accounts for more than one-quarter of the variances in their respective latent constructs. Additionally, from the AVEs, the collective items for the various sub-domains explained more than half of the variability in the target domain (cultural mix coping). Variances accounted for by both (a) individual items and (b) the collective items, met the minimum recommendations and provides high evidence of internal structure [62,63]. 

The results from the AVEs and HTMT showed that convergent and divergent sources of validity evidence were established using this sample. Evidence from the McDonald’s Omega coefficients also suggested high internal consistency among the items for their respective sub-domains. There were also relationships among the sub-domains. Summarily, the cultural mix coping inventory demonstrated sufficient evidence of internal structure. The implication is that relationships among the items and sub-domains support the meaningful definition of coping as measured by those proxies among healthcare professionals in Ghana.

Given that there could be a possible influence of the sex of individuals on how they cope with stress [68,69,70], an evaluation of the measurement invariance was conducted. However, no evidence of sex variance was found except for residual invariance, which supports the findings of the original version [46]. For the residual invariance, Brown [71] noted that such an outcome should not be an issue of concern since it is impossible to attain residual invariance, making it optional. Therefore, it can be said that there is evidence of item homogeneity for the cultural mix coping inventory.

For the usefulness of each item to the measure of the construct, the utility of item ESS4 was examined because of its loading below 0.50. A new model was tested with item ESS4 deleted (Model 2), relative to the original 16-item model (Model 1). Comparatively, the original 16-item model appeared to show a better fit than the 15-item model based on the fit indices. Notably, from the information criteria, the original 16-item model appeared to be better than the 15-item model. By implication, the 16-item model has minimal predictive error relative to the 15-item model. Hence, it is more precise in the measurement of coping than the 15-item. The original 16-item model is of high quality and produces more information about coping strategies than the 15-item model, confirming the results of the original version [46,50,51]. Further investigation is needed to explain the low factor loading of item ESS4 considering its utility to the measure. 

Considering item ESS4 (I learn to live with the stressor) and its sub-domain, one is likely to question the explicit contribution of that item to the emotional support sub-domain. Learning to live with a stressor may not necessarily be emotional support. Even though not established, this item could fall under other sub-domains of the cultural mix inventory. For instance, a healthcare professional might learn to live with a stressor by using either religious coping, behaviour disengagement or emotional coping. The emphasis is on learning to live with the stressor. Relatively, looking at the other items under the emotional support sub-dimension, it is worth noting that ‘the support’ is derived from someone, relatives or friends. However, item ESS4 was not referenced to any individual. This omission might have accounted for its low factor loading. Possibly, more description could be added to the item to make it reflect the meaning of the sub-domain (emotional support). For example, it may read: I learn to live with the stressor ‘by discussing the stressor with friends or relatives.

One of the objectives of the study was to assess the validity evidence based on external structure. Consequently, each of the four dimensions of the coping inventory was related to an anxiety measure. All the dimensions were negatively related to anxiety, except behaviour disengagement, which was positively related. These outcomes mean that coping as measured by the cultural mix coping inventory could highly predict anxiety, which is a different construct but related to coping. This further supports evidence of external structure [57]. The outcome that adaptive coping strategies (i.e., active coping and emotional support) are negatively associated with anxiety and maladaptive coping (i.e., behaviour disengagement) is positively correlated with anxiety [48,50,72] is an indication of sufficient validity of the coping inventory.

### 4.1. Limitations and Future Directions

The research was conducted among health professionals who encountered stressful situations from the onset and during the COVID-19 period. It is possible that the conditions that triggered the reported stressful experiences have changed. Therefore, further studies are required on the adaptation and re-validation of the coping inventory regarding the reproducibility of the instrument. These proposed studies may help to establish the applicability of the inventory among other working populations. The use of an online survey may result in recruiting healthcare professionals with heterogenous characteristics that may not be representative of healthcare workers in the country. 

Additionally, this study calls for further validation studies by conducting a bifactor CFA analysis, which has the capacity to investigate the relative contributions of an item to its sub-dimension as well as the general factor. This is important because one of the items had low factor loading although further evidence showed that the item was still relevant in contributing to the measurement of coping. What is unclear is whether that item belongs to the emotional support sub-domain; a piece of information that the bifactor CFA could provide. 

This study treated the four-point Likert scale as continuous, and as such, the maximum likelihood estimator was applied. The authors acknowledge that there is a long-standing debate regarding whether the Likert scale is considered interval or ordinal and the resulting implications on parameter estimation [73]. Hence, the use of a maximum likelihood estimator might affect the estimation of the parameters in this study. It is recommended that future validation studies should use other estimators, such as unweighted least squares and diagonally weighted least squares.

### 4.2. Practical Implications

The cultural mix coping inventory for stressful situations provides an accurate measure of coping strategies adopted by healthcare professionals in Ghana. Based on the findings of the study, future scholars in psychology-related areas can adopt/adapt this inventory for use in their research. Researchers who seek to assess the efficacy of some intervention programmes will find this inventory useful in scaling health workers into their various coping dimensions on the inventory. Hospital administrators can adopt this inventory to identify health workers who are utilizing dysfunctional coping mechanisms for appropriate client-specific interventions.

## 5. Conclusions

The findings of this study support the applicability and reproducibility of the cultural mix coping inventory for stressful situations among healthcare professionals. The findings confirmed the four-factor structure of the coping measure, with the items showing adequate construct validity and validity evidence based on the external structure of the measure. This inventory provides a useful and sound measure of coping among these health professionals.

## Figures and Tables

**Table 1:** The socio-demographic information of the participants.

Variable	Levels	Frequency	Percent
Sex	Male	114	36.5
	Female	198	63.5
Religion	Christian	286	91.7
	Muslim	24	7.7
	Atheist	2	0.6
Age range	20–24 years	10	3.2
	25–29 years	64	20.5
	30–34 years	108	34.6
	35–39 years	40	12.8
	40 years+	90	28.8
Education qualification	Certificate	26	8.3
	Diploma	90	28.8
	Bachelor’s	98	31.4
	Master’s	82	26.3
	PhD	16	5.1
Job designation	General nurse	216	69.2
	Psychologist	22	7.1
	Physician Assistant	16	5.1
	Surgical Doctors	30	9.6
	Midwife	28	9.0
Years of working	<1 year	32	10.3
	1–2 years	52	16.7
	3–4 years	36	11.5
	5 years+	192	61.5

**Table 2:** The mean, variance, skewness and kurtosis of the items.

Label	Items	Mean	Var.	Skew.	Kurt.
ACP1	I concentrate my effort on doing something about it	2.026	0.657	−0.444	−0.444
ACP2	I take additional action to try to get rid of the problem	2.038	0.644	−0.479	−0.340
ACP3	I take direct action to get around the stressor	1.866	0.762	−0.344	−0.613
ACP4	I do what has to be done, one step at a time	2.212	0.569	−0.505	−0.655
REL1	I put my trust in God/object of worship	2.211	0.850	−1.093	0.361
REL2	I seek help from my object of worship	1.821	1.240	−0.492	−1.115
REL3	I try to find comfort in my object of worship	1.930	1.043	−0.563	−0.838
REL4	I pray more than usual for my God to guard me	1.700	1.086	−0.344	−1.042
BEH1	I admit to myself that I can’t deal with the stressor and quit trying	0.633	0.910	1.235	0.184
BEH2	I just give up trying to reach my goal because of the stressor	0.550	0.835	1.515	1.061
BEH3	I give up the attempt in dealing with the stressor	0.479	0.633	1.519	1.247
BEH4	I reduce the amount of effort I’m putting into solving the problem	0.550	0.759	1.477	1.119
ESS1	I discuss how I feel about the stressor with someone	1.802	0.791	−0.393	−0.540
ESS2	I try to get emotional support from friends or relatives when dealing with the stressor	1.604	0.910	−0.133	−0.907
ESS3	I get sympathy and understanding from someone to reduce my fears about the stressor	1.553	0.969	−0.158	−0.989
ESS4	I learn to live with the stressor	1.891	1.011	−0.595	−0.704

Label—Abbreviation (short name) for the items.

**Table 3:** Model Fit Indices for the one-factor, three-factor, four-factor and second-order CFA models.

Indicators	One-Factor	Three-Factor	Four-Factor	Second-Order CFA
Chi-square	1676 *	992 *	148.388 *	2427.883 *
Degree of freedom	104	101	98	120
CMIN/Df (Minimum Discrepancy Function/Degrees of Freedom)	16.115	9.822	1.514	20.232
Comparative Fit Index (CFI)	0.321	0.615	0.954	0.426
Standard Root Mean Square of Residual (SRMR)	0.182	0.152	0.061	0.238
Root Mean Square Error of Approximation (RMSEA)	0.220	0.168	0.048	0.249
Akaike’s Information Criteria (AIC)	12,566	11,888	373.734	2459.883
Bayesian Information Criteria (BIC)	12,746	12,079	515.968	2519.771

* chi-square test significant at *p* < 0.001.

**Table 4:** Factor Loadings, AVE, HTMT and Reliability Estimates.

Dimensions	Label	Loading	*p*-Value *	AVE	Omega ω	Inter- Dimensions	HTMT Values
Active Coping (ACP)	ACP1	0.819	<0.001	0.742	0.830	ACP~RCP	0.952
	ACP2	0.769	<0.001			ACP~BEH	0.901
	ACP3	0.660	<0.001			ACP~ESS	0.937
	ACP4	0.721	<0.001			RCP~BEH	0.911
Religious Coping (RCP)	REL1	0.759	<0.001	0.762	0.846	RCP~ESS	0.963
	REL2	0.751	<0.001			BEH~ESS	0.906
	REL3	0.868	<0.001				
	REL4	0.668	<0.001				
Behaviour Disengagement (BEH)	BEH1	0.667	<0.001	0.800	0.873		
	BEH2	0.863	<0.001				
	BEH3	0.863	<0.001				
	BEH4	0.806	<0.001				
Emotional Support (ESS)	ESS1	0.580	<0.001	0.666	0.770		
	ESS2	0.878	<0.001				
	ESS3	0.832	<0.001				
	ESS4	0.373	<0.001				

* Significant at *p* < 0.001; HTMT—Heterotrait –monotrait (HTMT) Ratio of Correlation; and AVE—Average Variance Extracted.

**Table 5:** Model Fit Indices for Model 1 and Model 2.

Index	Model 1 (Four-Factor, 16 Items)	Model 2 (Four-Factor, 15 Items)
CMIN (Minimum Discrepancy Function)	148.388 *	237.888 *
DF (Degrees of Freedom)	98	84
CMIN/DF	1.514	2.832
CFI	0.954	0.931
SRMR	0.061	0.060
RMSEA	0.048	0.077
AIC	224.388	309.888
BIC	340.283	444.636

* Chi-square difference = 89.50, df = 14, *p* < 0.001.

**Table 6:** Measurement Invariance based on the Sex of the Participants.

Model Level Invariance			Multi-Group Analysis				
Fit Indices	Male	Female	Model	χ^2^	df	χ^2^/df	*p*
Chi-square, χ^2^ (df)	269.210 * (91)	240.479 * (91)	Measurement weights	30.331	24	1.263	0.174
CMIN	2.958	2.643	Structural covariances	74.279	44	1.688	0.063
GFI	0.943	0.951	Measurement residuals	192.955	76	2.539	0.000
SRMR	0.061	0.061					
RMSEA	0.063	0.064					
CFI	0.949	0.948					

Difference in Unconstrained vs. Fully Constrained: *χ*^2^ (31) = 11.012, *p* = 0.116; * Chi-square test is significant at *p* < 0.001.

**Table 7:** The relationships between Coping Measures and Anxiety Measures and Descriptive Statistics.

Dimensions	ACP	RCP	BEH	ESS	AXT
Active coping (ACP)	1				
Religious coping (RCP)	−0.289 **	1			
Behaviour disengagement (BEH)	−0.660 **	0.316 **	1		
Emotional support (ESS)	0.371 **	0.302 **	−0.218 **	1	
Anxiety (AXT)	−0.807 **	−0.554 **	0.647 **	−0.607 **	1
Mean values	2.042	1.921	0.554	1.718	2.327
Standard deviation	0.655	0.844	0.754	0.484	0.690

** Correlation is significant at the 0.01 level (two-tailed).

## Data Availability

Anonymized data is available upon reasonable request through the corresponding author.

## References

[1] Couarraze S., Delamarre L., Marhar F., Quach B., Jiao J., Avilés Dorlhiac R., Saadaoui F., Liu A.S., Dubuis B., Antunes S. (2021). The major worldwide stress of healthcare professionals during the first wave of the COVID-19 pandemic–the international COVISTRESS survey. PLoS ONE.

[2] Odonkor S.T., Adams S. (2021). Predictors of stress and associated factors among healthcare workers in Western Ghana. Heliyon.

[3] Yehya A., Sankaranarayanan A., Alkhal A., Al Naemi H., Almeer N., Khan A., Ghuloum S. (2020). Job satisfaction and stress among healthcare workers in public hospitals in Qatar. Arch. Environ. Occup. Health.

[4] Cabarkapa S., King J.A., Ng C.H. (2020). The psychiatric impact of COVID-19 on healthcare workers. Aust. J. Gen. Pract..

[5] Saragih I.D., Tonapa S.I., Saragih I.S., Advani S., Batubara S.O., Suarilah I., Lin C.J. (2021). Global prevalence of mental health problems among healthcare workers during the COVID-19 pandemic: A systematic review and meta-analysis. Int. J. Nurs. Stud..

[6] Teo I., Chay J., Cheung Y.B., Sung S.C., Tewani K.G., Yeo L.F., Yang G.M., Pan F.T., Ng J.Y., Abu Bakar Aloweni F. (2021). Healthcare worker stress, anxiety and burnout during the COVID-19 pandemic in Singapore: A 6-month multi-centre prospective study. PLoS ONE.

[7] Zhang Y., Wang C., Pan W., Zheng J., Gao J., Huang X., Cai S., Zhai Y., Latour J.M., Zhu C. (2020). Stress, burnout, and coping strategies of frontline nurses during the COVID-19 epidemic in Wuhan and Shanghai, China. Front. Psychiatry.

[8] Jones G., Hocine M., Salomon J., Dab W., Temime L. (2015). Demographic and occupational predictors of stress and fatigue in French intensive-care registered nurses and nurses’ aides: A cross-sectional study. Int. J. Nurs. Stud..

[9] Tsai Y.C., Liu C.H. (2012). Factors and symptoms associated with work stress and health-promoting lifestyles among hospital staff: A pilot study in Taiwan. BMC Health Serv. Res..

[10] Aly H.M., Nemr N.A., Kishk R.M., Abu Bakr Elsaid N.M. (2021). Stress, anxiety and depression among healthcare workers facing COVID-19 pandemic in Egypt: A cross-sectional online-based study. BMJ Open.

[11] Lenzo V., Quattropani M.C., Sardella A., Martino G., Bonanno G.A. (2021). Depression, anxiety, and stress among healthcare workers during the COVID-19 outbreak and relationships with expressive flexibility and context sensitivity. Front. Psychol..

[12] Xiang Y.T., Yang Y., Li W., Zhang L., Zhang Q., Cheung T., Ng C.H. (2020). Timely mental health care for the 2019 novel coronavirus outbreak is urgently needed. Lancet Psychiatry.

[13] LeBlanc V.R. (2009). The effects of acute stress on performance: Implications for health professions education. Acad. Med..

[14] Koinis A., Giannou V., Drantaki V., Angelaina S., Stratou E., Saridi M. (2015). The impact of healthcare workers job environment on their mental-emotional health. Coping Strateg. Case A Local Gen. Hosp. Health Psychol. Res..

[15] Kushnir T., Greenberg D., Madjar N., Hadari I., Yermiahu Y., Bachner Y.G. (2014). Is burnout associated with referral rates among primary care physicians in community clinics?. Fam. Pract..

[16] Panagioti M., Geraghty K., Johnson J., Zhou A., Panagopoulou E., Chew-Graham C., Peters D., Hodkinson A., Riley R., Esmail A. (2018). Association between physician burnout and patient safety, professionalism, and patient satisfaction: A systematic review and meta-analysis. JAMA Intern. Med..

[17] Lazarus R.S. (1993). Coping theory and research: Past, present, and future. Psychosom. Med..

[18] Lazarus R.S. (2013). Fifty Years of the Research and Theory of RS Lazarus: An Analysis of Historical and Perennial Issues.

[19] Folkman S., Lazarus R.S. (1980). An analysis of coping in a middle-aged community sample. J. Health Soc. Behav..

[20] Ibrahim N., Ong H.C., Wahab S. (2017). Development and validation of a coping scale for caregivers in Malaysia. Malays. J. Med. Sci..

[21] Magliano L., Guarneri M., Marasco C., Tosini P., Morosini P.L., Maj M. (1996). A new questionnaire assessing coping strategies in relatives of patients with schizophrenia: Development and factor analysis. Acta Psychiatr. Scand..

[22] Alonso-Tapia J., Rodríguez-Rey R., Garrido-Hernansaiz H., Ruiz M., Nieto C. (2016). Coping assessment from the perspective of the person-situation interaction: Development and validation of the Situated Coping Questionnaire for Adults (SCQA). Psicothema.

[23] Finset A., Steine S., Haugli L., Steen E., Laerum E. (2002). The brief approach/avoidance coping questionnaire: Development and validation. Psychol. Health Med..

[24] Gaudreau P., Blondin J.P. (2002). Development of a questionnaire for the assessment of coping strategies employed by athletes in competitive sport settings. Psychol. Sport Exerc..

[25] DanIsman I.G., Yıldız N., Yigit I. (2017). Development of a coping with stress scale for a non-western population of children and adolescents. Anxiety Stress Coping.

[26] Kowalski K.C., Crocker P.R.E. (2001). Development and validation of coping function questionnaire for adolescents in sport. J. Sport Exerc. Psychol..

[27] Boujut E., Bruchon-Schweitzer M., Dombrowski S. (2012). Coping among students: Development and validation of an exploratory measure. Psychology.

[28] Côté L., Lauzier M., Beauchamp G., Guertin F. (2018). Development and validation of an instrument measuring the coping strategies in situations of stress. Int. J. Psychol. Behav. Sci..

[29] Kato T. (2013). Assessing coping with interpersonal stress: Development and validation of the interpersonal stress coping scale in Japan. Int. Perspect. Psychol. Res. Pract. Consult..

[30] Roger D., Jarvis G., Najarian B. (1993). Detachment and coping: The construction and validation of a new scale for measuring coping strategies. Personal. Individ. Differ..

[31] Hammer A.L., Marting M.S. (1988). Manual for the Coping Resources Inventory.

[32] Carver C.S., Scheier M.F., Weintraub J.K. (1989). Assessing coping strategies: A theoretically based approach. J. Personal. Soc. Psychol..

[33] Endler N.S., Parker J.D. (1990). Multidimensional assessment of coping: A critical evaluation. J. Personal. Soc. Psychol..

[34] Boudreaux E., Catz S., Ryan L., Amaral-Melendez M., Brantley P.J. (1995). The ways of religious coping scale: Reliability, validity, and scale development. Assessment.

[35] Pargament K.I. (1999). Religious/spiritual coping. Multidimensional Measurement of Religiousness/Spirituality for Use in Health Research.

[36] Pargament K.I., Koenig H.G., Perez L.M. (2000). The many methods of religious coping: Development and initial validation of the RCOPE. J. Clin. Psychol..

[37] Pargament K., Feuille M., Burdzy D. (2011). The Brief RCOPE: Current psychometric status of a short measure of religious coping. Religion.

[38] Frías M.T., Shaver P.R., Díaz-Loving R. (2014). Individualism and collectivism as moderators of the association between attachment insecurities, coping, and social support. J. Soc. Pers. Relatsh..

[39] Glazer S. (2006). Social support across cultures. Int. J. Intercult. Relat..

[40] Ndemanu M.T. (2018). Traditional African religions and their influences on the worldviews of Bangwa people of Cameroon: Expanding the cultural horizons of study abroad students and professionals. Front. Interdiscip. J. Study Abroad.

[41] Shekriladze I., Javakhishvili N., Chkhaidze N. (2021). Culture Related Factors May Shape Coping During Pandemics. Front. Psychol..

[42] Schwartz S.H., Vinken H., Soeters J., Ester P. (2004). Mapping and interpreting cultural differences around the world. Comparing Cultures, Dimensions of Culture in a Comparative Perspective.

[43] Taylor S.E., Sherman D.K., Kim H.S., Jarcho J., Takagi K., Dunagan M.S. (2004). Culture and social support: Who seeks it and why?. J. Personal. Soc. Psychol..

[44] Guan Y., Deng H., Zhou X. (2020). Understanding the impact of the COVID-19 pandemic on career development: Insights from cultural psychology. J. Vocat. Behav..

[45] Croucher S.M., Zeng C., Rahmani D., Sommier M. (2017). Religion, culture, and communication. Oxford Research Encyclopedia of Communication.

[46] Quansah F., Ankomah F., Hagan J.E., Srem-Sai M., Frimpong J.B., Sambah F., Schack T. (2022). Development and validation of an inventory for stressful situations in university students involving coping mechanisms: An interesting cultural mix in Ghana. Psych.

[47] Frimpong J.B., Agormedah E.K., Srem-Sai M., Quansah F., Hagan J.E. (2022). Examining Risk Perception and Coping Strategies of Senior High School Teachers in Ghana: Does COVID-19-Related Knowledge Matter?. COVID.

[48] Hagan J.E., Quansah F., Frimpong J.B., Ankomah F., Srem-Sai M., Schack T. (2022). Gender risk perception and coping mechanisms among Ghanaian university students during the COVID-19 pandemic. Healthcare.

[49] Hagan J.E., Quansah F., Ankomah F., Agormedah E.K., Srem-Sai M., Frimpong J.B., Schack T. (2022). Linking COVID-19-related awareness and anxiety as determinants of coping strategies’ utilization among senior high school teachers in Cape Coast Metropolis, Ghana. Soc. Sci..

[50] Quansah F., Frimpong J.B., Sambah F., Oduro P., Anin S.K., Srem-Sai M., Hagan J.E., Schack T. (2022). COVID-19 pandemic and teachers’ classroom safety perception, anxiety and coping strategies during instructional delivery. Healthcare.

[51] Quansah F., Hagan J.E., Ankomah F., Srem-Sai M., Frimpong J.B., Sambah F., Schack T. (2022). Relationship between COVID-19 related knowledge and anxiety among university students: Exploring the moderating roles of school climate and coping strategies. Front. Psychol..

[52] Poku C.A., Donkor E., Naab F. (2020). Determinants of emotional exhaustion among nursing workforce in urban Ghana: A cross-sectional study. BMC Nurs..

[53] Kaburi B.B., Bio F.Y., Kubio C., Ameme D.K., Kenu E., Sackey S.O., Afari E.A. (2019). Psychological working conditions and predictors of occupational stress among nurses, Salaga Government Hospital, Ghana, 2016. Pan Afr. Med. J..

[54] Afulani P.A., Nutor J.J., Agbadi P., Gyamerah A.O., Musana J., Aborigo R.A., Odiase O., Getahun M., Ongeri L., Malechi H. (2021). Job satisfaction among healthcare workers in Ghana and Kenya during the COVID-19 pandemic: Role of perceived preparedness, stress, and burnout. PLOS Glob. Public Health.

[55] Afulani P.A., Gyamerah A.O., Nutor J.J., Laar A., Aborigo R.A., Malechi H., Sterling M., Awoonor-Williams J.K. (2021). Inadequate preparedness for response to COVID-19 is associated with stress and burnout among healthcare workers in Ghana. PLoS ONE.

[56] Ofori A.A., Osarfo J., Agbeno E.K., Manu D.O., Amoah E. (2021). Psychological impact of COVID-19 on health workers in Ghana: A multicentre, cross-sectional study. SAGE Open Med..

[57] American Educational Research Association (AERA), American Psychological Association (APA), National Council on Measurement in Education (NCME) (2011). Standards for Educational and Psychological Testing.

[58] Kane M. (2012). Validating score interpretations and uses. Lang. Test..

[59] Tabachnick B., Fidell L. (2013). Using Multivariate Statistics.

[60] Beck A.T., Epstein N., Steer R.A. (1988). An inventory for measuring clinical anxiety: Psychometric properties. J. Consult. Clin. Psychol..

[61] Hagan J.E., Quansah F., Anin S.K., Sorkpor R.S., Abieraba R.S.K., Frimpong J.B., Srem-Sai M., Schack T. (2022). COVID-19-Related Knowledge and Anxiety Response among Physical Education Teachers during Practical In-Person Lessons: Effects of Potential Moderators. Behav. Sci..

[62] DeVellis R.F. (2017). Scale Development: Theory and Applications.

[63] Fornell C., Larcker D. (1981). Evaluating structural equation models with unobserved variables and measurement error. J. Mark. Res..

[64] Hensler J., Ringle C.M., Sarstedt M. (2015). A new criterion for assessing discriminant validity in variance-based structural equation modeling. J. Acad. Mark. Sci..

[65] McDonald R.P. (1999). Test Theory: A Unified Treatment.

[66] Hayes A.F., Coutts J.J. (2020). Use omega rather than Cronbach’s alpha for estimating reliability. Commun. Methods Meas..

[67] Hair J.F., Black W.C., Babin B.J., Anderson R.E. (2010). Multivariate Data Analysis.

[68] Graves B.S., Hall M.E., Dias-Karch C., Haischer M.H., Apter C. (2021). Gender differences in perceived stress and coping among college students. PLoS ONE.

[69] Pinto A., Pasian S.R., Malloy-Diniz L.F. (2021). Gender invariance and psychometric properties of a Brazilian version of the Emotion Regulation Questionnaire (ERQ). Trends Psychiatry Psychother..

[70] Pulido-Martos M., Fernández-Sánchez M.D., Lopez-Zafra E. (2020). Measurement invariance across gender and age in the Connor–Davidson Resilience Scale (CD-RISC) in a Spanish general population. Qual. Life Res..

[71] Brown T.A. (2015). Confirmatory Factor Analysis for Applied Research.

[72] Weston D., Ip A., Amlôt R. (2020). Examining the application of behaviour change theories in the context of infectious disease outbreaks and emergency response: A review of reviews. BMC Public Health.

[73] Carifio L., Perla R. (2008). Resolving the 50-year debate around using and misusing Likert scales. Med. Educ..

